# Impact of socioeconomic risk factors on the seroprevalence of cytomegalovirus infections in a cohort of pregnant Polish women between 2010 and 2011

**DOI:** 10.1007/s10096-014-2170-3

**Published:** 2014-06-06

**Authors:** W. Wujcicka, Z. Gaj, J. Wilczyński, W. Sobala, E. Śpiewak, D. Nowakowska

**Affiliations:** 1 Department of Fetal-Maternal Medicine and Gynecology, Polish Mother’s Memorial Hospital Research Institute, 281/289 Rzgowska Street, Lodz, 93-338 Poland; 2 Department of Fetal-Maternal Medicine and Gynecology, Third Chair of Gynecology and Obstetrics, Medical University of Lodz, 281/289 Rzgowska Street, Lodz, 93-338 Poland; 3 Department of Environmental Epidemiology, Institute of Occupational Medicine, Lodz, Poland; 4 Department of Microbiology, Polish Mother’s Memorial Hospital Research Institute, Lodz, Poland

## Abstract

The purpose of this investigation was to perform an evaluation of the prevalence and socioeconomic risk factors for human cytomegalovirus (HCMV) infections in a cohort of Polish pregnant women between 2010 and 2011. HCMV-specific IgG and IgM antibody levels were assayed with enzyme-linked immunosorbent assay (ELISA) tests in serum samples collected from 1,250 pregnant women attending outpatient obstetric clinics and hospitalized at two hospitals in Lodz. The seroprevalence of anti-HCMV IgG and IgM antibodies was 62.4 and 2.2 %, respectively, and differed significantly between age-stratified groups (*p* ≤ 0.05). The highest IgG prevalence was observed in women above 36 years of age (76.2 %) and IgM in adolescent women aged 16–20 years (6.0 %). Of the various socioeconomic factors, age above 36 years, basic and professional education, and offspring were significantly associated with HCMV IgG prevalence rates (PRs; 1.89, 1.80, and 1.56, respectively). Financial status, occupational risk related to contact with children, and transfusions were not related to the prevalence of IgG antibodies. The IgM prevalence was not associated with any of the analyzed risk factors. A slightly higher prevalence was observed in women who were transfused in the past, but the relationship was not significant. The current data have revealed a decrease in HCMV IgG seroprevalence in our region during recent years (62.4 vs. 76.7 %). Basic and professional education, as well as bringing up offspring, were determined as significant risk factors for HCMV infections in Polish pregnant women [risk ratio (RR) 1.20 and 1.17, respectively], suggesting that the primary and secondary prophylaxis of cytomegaly is necessary during pregnancy, even if screening is not mandatory.

## Introduction

Human cytomegalovirus (HCMV) is the most common factor of intrauterine viral infections, transmitted in urine, blood, saliva, by breastfeeding, genitourinary tract secretions, feces, tears, and transplanted organs [1–7]. HCMV infections may be acquired prenatally, perinatally, or postnatally, and can cause permanent physical sequelae, with an increased risk of infant mortality. Congenital infections occur via vertical transmission of the virus by a hematogenous route from infected pregnant woman to the fetus via the placenta [6]. The incidence rates of viral transmissions from mothers with primary infections during pregnancy to their fetuses are estimated to be in the range 30–40 %, while in those with recurrent infections, the range is 0.2–2.2 % [8–12]. The diagnosis of cytomegaly is based mostly on serological tests during pregnancy. The primary infection is defined as HCMV IgG seroconversion during pregnancy but, in most cases, the distinction between primary and non-primary maternal cytomegaly is very difficult, due to the lack of data on the preconception serologic status. The presence of specific HCMV IgM antibodies and the low IgG avidity do not always indicate recent primary infection [8, 13]. In most cases, systematic ultrasound is not sensitive enough to detect signs of fetal cytomegaly beside its most characteristic symptoms, such as microcephaly, ventriculomegaly, increased periventricular echogenicity, and calcifications [8, 14].

Congenital HCMV infections during the first trimester of pregnancy are more likely to cause a severe form of the disease, but symptomatic cases were also reported, when transmission occurred in the third trimester of pregnancy [15]. Approximately 10 to 15 % of in utero infected newborns demonstrate clinical symptoms observed in various organs and systems and 85–90 % of these children will develop some degree of psychomotor and mental retardation, including visual impairment and sensorineural hearing loss [8, 16]. Children born with asymptomatic HCMV infections (85–90 %) may also acquire cytomegaly-related symptoms, such as hearing impairment and difficulties in learning during the first months or, more often, in the first few years of life [6, 8, 11].

The prevalence of HCMV IgG antibodies varies between continents and countries, ranging from 40 to 100 % [7, 17, 18]. The prevalence rate of 76.7 % was observed in Polish pregnant women between the years 1999 and 2009, being one of the highest in Europe, alongside such countries as Sweden or Italy (72 and 68 %, respectively) [3, 17, 19]. So far, studies in different countries have revealed elevated prevalence rates of congenital HCMV, related to non-white race, increased sexual activity with multiple partners, age below 25 years, an increased age of pregnant women, multiparity, preschool children in the household, and occupational exposure to children, as well as lower socioeconomic status (SES) [7, 20–22]. The reported SES risk factors for increased HCMV prevalence included a lower level of education and lower incomes of pregnant women [23–26].

In the reported study, we investigated the prevalence of specific anti-HCMV IgG and IgM antibodies and the socioeconomic risk factors of HCMV infections in a group of pregnant women from Poland attending outpatient obstetric clinics and hospitalized between 2010 and 2011 at the Polish Mother’s Memorial Hospital Research Institute (PMMHRI) and at the Ludwig Rydygier Hospital in Lodz.

## Materials and methods

The study included 1,250 randomly selected, newly registered pregnant women who attended the outpatient obstetric clinics and were treated at the PMMHRI and the Ludwig Rydygier Hospital between April, 2010 and March, 2011. The cohort, hospitalized at the PMMHRI, consisted of pregnant women from the Lodz Province and from other Polish regions, as the PMMHRI houses a reference perinatal care center.

### Serological tests

Blood samples were obtained from pregnant women twice during pregnancy (at the 12th–15th and the 30th–34th gestational weeks) and within a day from childbirth. Blood specimens of 2.6 ml were collected from participants, who signed an informed consent form. The blood was collected into sterile, nonanticoagulated tubes. The collected samples were centrifuged at 3,000 × g for 10 min and serum fractions were stored at −20 °C.

Serum anti-HCMV IgG and IgM antibody levels were assayed by enzyme-linked immunosorbent assay (ELISA) tests (LIAISON®, DiaSorin, Italy), and seropositivity was determined, using the manufacturer’s guidelines. The screening was performed using a LIAISON® immunoassay analyzer. All samples were considered as IgG- or IgM-positive when the antibody levels were >0.4 IU/ml and >30 AU/ml, respectively. For IgG avidity assessment, the indexes <0.300 were interpreted as low avidity suggesting recent infection, whereas the indexes ≥0.300 were interpreted as high avidity. Pregnant women were considered as probably recently infected in cases where specific IgG were elevated, IgM were present, and IgG avidity was low. The kinetics of the specific antibodies was of great importance. In those women, the presence of HCMV DNA was checked using a real-time Q PCR assay for the viral *UL55* gene in blood, urine, and amniotic fluid specimens [4]. DNA isolation and real-time Q PCR were carried out at the Laboratory of Molecular Virology and Biological Chemistry, Institute of Medical Biology, Polish Academy of Sciences in Lodz.

All participants completed a structured questionnaire, including data on the demographic and socioeconomic status, as well as previous exposure to blood transfusion. The pregnant women subjectively classified their financial status into one of four categories: bad, average, good, and very good. The study had been approved by the Ethical Committee of the PMMHRI in Lodz and all the women participating in the study signed the consent form.

### Statistical analysis

The seroprevalence rates of anti-HCMV IgG and IgM antibodies were assessed by means of descriptive statistics. Relationships were determined between the prevalence rates of HCMV and various socioeconomic factors, including age, level of education, offspring, financial status, and a risk of occupational contact with children and transfusions, using cross-tabulation and Pearson’s Chi-squared test. Yates’ continuity correction for the Chi-squared test was used to determine differences in the risk of HCMV infections between pregnant women with and without children at home. Fisher’s exact test for count data was used to determine the significance of the differences in HCMV IgM prevalence rates among pregnant women with different socioeconomic status. For all socioeconomic factors, the prevalence rates and risk ratios (PRs and RRs, respectively) of HCMV IgG were assessed, using a binary logistic regression model. All results were determined as being statistically significant at the significance level of *p* ≤ 0.05. Data were analyzed using the Stata v.11 software (StataCorp, College Station, TX, USA).

## Results

### Prevalence of IgG antibodies in different age groups

The study cohort consisted of 1,250 pregnant women, aged 16–45 years, among whom 62.4 % (780/1,250) were determined as HCMV IgG seropositive (see Table 1). The total population of women was grouped into five age classes. The prevalence rate was significantly associated with the age of the patients (*p* = 0.0069), with the highest value of 76.2 % (99/130) observed in pregnant women aged ≥36 years, and the lowest prevalence rate of 58.5 % (298/509) in women aged 26–30 years. A slightly higher prevalence rate of 66.0 % (33/50) was found in patients aged 16–20 years. A significant association between HCMV prevalence and age was observed among pregnant women above 36 years of age [PR = 1.89; 95 % confidence interval (CI) 1.17–3.07].

**Table 1:** Risk factors for human cytomegalovirus (HCMV) IgG prevalence in different socioeconomic groups of pregnant women

Risk factors	Classes	No. tested (%)	HCMV IgG positive (%)	χ^2^	*p*-Value^a^	PR	95 % CI	*p*-Value^a^	RR	95 % CI	*p*-Value^a^
Total		1,250 (100 %)	780 (62.4 %)								
Age	≤20	50 (4.0 %)	33 (66.0 %)	14.13	0.0069	1.00	0.51–1.98	0.9917	1.01	0.80–1.29	0.0910
	21–25	199 (15.9 %)	122 (61.3 %)			1.00	0.69–1.46	0.9986	1.01	0.88–1.16	
	26–30	509 (40.7 %)	298 (58.5 %)			1.0			1.0	Ref.	
	31–35	362 (29.0 %)	228 (63.0 %)			0.99	0.73–1.33	0.9235	1.00	0.90–1.22	
	≥36	130 (10.4 %)	99 (76.2 %)			1.89	1.17–3.07	0.0095	1.20	1.06–1.36	
Education	Higher	667 (56.5 %)	387 (58.0 %)	12.72	0.0017	1.0			1.0	Ref.	0.0276
	Secondary	369 (31.3 %)	238 (64.5 %)			1.34	1.00–1.79	0.0496	1.10	0.99–1.22	
	Primary and vocational	144 (12.2 %)	105 (72.9 %)			1.80	1.14–2.83	0.0116	1.20	1.05–1.37	
Having children	No	691 (59.1 %)	396 (57.3 %)	17.45^b^	≤0.0001	1.0			1.0	Ref.	0.0016
	Yes	479 (40.9 %)	333 (69.5 %)			1.56	1.19–2.05	0.0012	1.17	1.06–1.29	
Financial status	Bad	48 (3.8 %)	28 (58.3 %)	3.28	0.5115	Not calculated^c^			1.0	Ref.	0.3469
	Average	359 (28.7 %)	228 (63.5 %)			–			1.10	0.87–1.40	
	Good	616 (49.3 %)	391 (63.5 %)			–			1.09	0.85–1.38	
	Very good	76 (6.1 %)	41 (53.9 %)			–			0.94	0.68–1.29	
	Unknown	151 (12.1 %)	92 (60.9 %)			–			0.95	0.71–1.28	
Risk of occupational contact with children	No	799 (63.3 %)	491 (61.5 %)	1.45	0.4843	–			1.0	Ref.	0.6520
	Yes	373 (29.8 %)	236 (63.3 %)			Not calculated^c^			1.02	0.93–1.12	
	Unknown	78 (6.2 %)	53 (67.9 %)			–			1.21	0.85–1.72	
Transfusions	No	1,134 (96.1 %)	707 (62.3 %)	0.64	0.4247	1.0			1.0	Ref.	0.2004
	Yes	46 (3.9 %)	26 (56.5 %)			0.56	0.30–1.06	0.0747	0.85	0.67–1.09	

*No. tested* number of tested pregnant women, *Ref.* reference class, *PR* prevalence ratio, *95 % CI* 95 % confidence interval, *RR* risk ratio

^a^ *p* ≤ 0.05 is considered as significant

^b^Pearson’s Chi-squared test with Yates’ continuity correction

^c^Not calculated since RR was assessed as a more adequate index

### IgG prevalence in various socioeconomic groups

The study population was classified into three groups, according to the education level (see Table 1). Appropriate data were obtained for 1,180 pregnant women. Higher education was recorded in 56.5 % (667/1,180) of women, secondary education in 31.3 % (369/1,180), and primary and vocational education was reported by 12.2 % (144/1,180). The HCMV prevalence rate differed significantly among particular groups with various education levels (*p* = 0.0017). The prevalence rate decreased with increasing education level, ranging from 72.9 % (105/144) in the group with primary and vocational education to 58.0 % (387/667) in the group with university education. A significant association with the prevalence rate of infection was observed for secondary, primary, and vocational education (PR = 1.34; 95 % CI 1.00–1.79 and PR = 1.80; 95 % CI 1.14–2.83, respectively).

The group of 1,170 pregnant women was also described in relation to offspring in the household (see Table 1). In the study population, 40.9 % (479/1,170) of pregnant women had children. The prevalence rate differed significantly among the groups of patients with and without children (*p* ≤ 0.0001). The differences stayed significant after Yates’ continuity correction. In women with offspring, the prevalence rate of infection was 1.56 times higher than in those without children: 69.5 % (333/479) vs. 57.3 % (396/691) (95 % CI 1.19–2.05; *p* = 0.0012). Additionally, the cohort was evaluated according to the financial status and risk of occupational contact with children, which was characteristic for professional groups, such as school teachers, health care workers, social and community workers, as well as sales staff. Neither the financial status nor the occupational contact with children and blood transfusions influenced the prevalence rate (*p* = 0.5115, *p* = 0.4843, and *p* = 0.4247, respectively; see Table 1). Considering the financial status, the highest prevalence of infection was observed among the pregnant women with average or good financial status (63.5 %), and the lowest prevalence rate among women with the best financial status (53.9 %). The prevalence rate among patients with or without the occupational risk related to contact with children was 63.3 % (236/373) and 61.5 % (491/799), respectively. Pregnant women with or without blood transfusions in their history had prevalence rates of 56.5 % (26/46) and 62.3 % (707/1,134), respectively. However, the observed differences were not significant.

Out of the various socioeconomic factors, the level of education and offspring in the household were determined as being significantly associated with HCMV infection rates (*p* = 0.0276 and *p* = 0.0016, respectively). The highest risk of infection was observed in pregnant women with primary and vocational education (RR 1.20, 95 % CI 1.05–1.37). A slightly lower (1.17) RR (95 % CI 1.06–1.29) was found in women with offspring. The age of pregnant women, their financial status, the risk of occupational contact with children, and blood transfusions in their history were not associated with HCMV infections (*p* = 0.0910, *p* = 0.3469, *p* = 0.6520, and *p* = 0.2004, respectively). However, the age of pregnant women tended to be an important risk factor of HCMV infections, with the highest RR observed in patients above 36 years of age (1.20, 95 % CI 1.06–1.36).

### Prevalence of IgM antibodies

The prevalence of IgM antibodies was 2.2 % (28/1,250; see Table 2) and, similarly to IgG, it was significantly associated with the patients’ age (*p* = 0.0174). The highest prevalence rate of 6.0 % (3/50) was observed in pregnant women at the age of 16–20 years, and a little lower (4.6 %) (6/130) in those aged ≥36 years. Similarly to IgG antibodies, the lowest prevalence rate of 1.0 % (5/509) was found in the group aged 26–30 years.

**Table 2:** HCMV IgM prevalence and risk factors in the studied cohort of pregnant women

Risk factors	Classes	HCMV IgM positive (%)
Total		28 (2.2 %)
Age	≤20	3 (6.0 %)
	21–25	4 (2.0 %)
	26–30	5 (1.0 %)
	31–35	10 (2.8 %)
	≥36	6 (4.6 %)
Education	Higher	14 (2.1 %)
	Secondary	7 (1.9 %)
	Primary and vocational	3 (2.1 %)
Having children	No	15 (2.2 %)
	Yes	10 (2.1 %)
Financial status	Bad	1 (2.1 %)
	Average	5 (1.4 %)
	Good	16 (2.6 %)
	Very good	2 (2.6 %)
	Unknown	4 (2.6 %)
Risk of occupational contact with children	No	16 (2.0 %)
	Yes	9 (2.4 %)
Transfusions	No	23 (2.0 %)
	Yes	2 (4.3 %)

### IgM prevalence in various socioeconomic groups

The prevalence of specific IgM in the study cohort reached 2.2 % and was independent of any of the analyzed socioeconomic factors. According to the level of education, similar prevalence rates (2.0 %) were observed (*p* = 1.0000; see Table 2). The prevalence rate of about 2.0 % was not correlated either with offspring or with the occupational risk of contact with children (*p* = 1.0000 and *p* = 0.4529, respectively). Taking into account the financial status, the prevalence rate of IgM varied from 1.4 to 2.6 % between classes, but the differences were not significant (*p* = 0.6863). Pregnant women with transfusions in their history presented with slightly higher prevalence rates than those without such events, but the relationship was non-significant [4.3 % (2/46) vs. 2.0 % (23/1,134); *p* = 0.2544)].

## Discussion

The seroprevalence of anti-HCMV IgG antibodies differs throughout the world, ranging from 40 to 100 % [7, 17, 18]. The highest prevalence rates were reported in South America, Africa, and Asia [6, 20]. In Europe, the lowest prevalence rates were shown for pregnant women in the Netherlands (41 %), followed by France (46 %) and the United Kingdom (49 %) [27–29]. Higher prevalence rates were observed in Belgium and Finland (50–60 %) [30, 31]. In the reported study, we observed a prevalence rate of 62.4 % in the cohort of 1,250 pregnant women. Given the prevalence rate of 76.7 % observed in Polish pregnant women hospitalized at the same department in the earlier period between 1999 and 2009, the current data revealed a decrease in the HCMV prevalence rate in the recent years [3], being similar to that for pregnant women in Spain (57 %), Norway (60 %), and Italy (68 %). Due to that reason, Poland can be placed among the European countries with moderate prevalence rates [17, 32, 33]. Higher prevalence values were observed in Turkey, ranging from 94.9 % in the South to 96.4 % in the North of the country [18, 34].

Evaluating the age of pregnant women in our study cohort, age above 36 years was the most important factor (PR = 1.89) associated with HCMV prevalence. A significantly higher prevalence rate was observed in women above 36 years old than in younger ones, aged 26–30 years (76.2 vs. 58.5 %). Similarly, higher prevalence rates for older pregnant women were observed in the British Isles, Italy, India, and Australia [23, 25, 26, 35].

In our study, the age of pregnant women also tended to be a risk factor of HCMV infections, with the highest risk ratio (RR = 1.20) observed in women older than 36 years. The lack of statistical significance for the relationship between the age of pregnant women and the prevalence rate of infection possibly resulted from the relatively small number of pregnant women above 36 years of age, compared to the number of women aged 26–30 and 31–35 years. By contrast, the reports from studies on Northern Italian pregnant women have indicated no association between HCMV prevalence and patient age [17], which might have resulted from the low number of pregnant women in the youngest and oldest study groups (1.7 and 3.2 % of the study cohort, respectively) [17]. In our study cohort, the prevalence rate in the youngest pregnant women aged 16–20 and 21–25 years was higher than that observed among women aged 26–30 years (66.0 and 61.3 % vs. 58.5 %, respectively). One possible explanation for the relatively high prevalence, observed in the youngest groups, could refer to the small number (4.0 and 15.9 % of the whole cohort, respectively). However, the young age of pregnant women below 25 years of age, also reported in other studies, seems to be an important reason as well [22, 28].

Beside the region and age, other socioeconomic risk factors for HCMV infections have also been reported [21, 24, 36]. Higher prevalence rates in correlation with lower socioeconomic status were determined in pregnant women from Finland (60.9 vs. 76.4 %) and India (63.4 vs. 96.9 %) [24, 25]. A significant influence of this factor on the prevalence rates of the infection was also observed in the British Isles and Italy [23, 26]. In our study cohort, we estimated a significant influence of the education level on the HCMV prevalence rate. The highest prevalence and risk of HCMV infection were characteristic for pregnant women with primary and vocational education (72.9 % vs. 64.5 and 58.0 % for primary and vocational vs. secondary and higher education, respectively; PR = 1.80, RR = 1.20). A slightly lower influence on the prevalence rate of HCMV infection was observed for women with secondary education (PR = 1.56). Since no other study has yet been performed to investigate the relationships between education level and the prevalence of HCMV, further research on the role of education as a risk factor of HCMV infections in pregnant women seems to be challenging and necessary.

HCMV prevalence among pregnant women might also be affected by having children [21, 25, 28]. In our study, we observed a significantly higher prevalence of HCMV infection in pregnant women with offspring than in those without children (69.5 % vs. 57.3; PR = 1.56). It is consistent with the differences in the prevalence rates reported for pregnant women from India (96.0, 93.1, and 66.7 % for women having ≥3, 1–2, and no children, respectively) and the British Isles (from 66.2 to 46.4 % vs. 38.9 % for women having 4–1 vs. those without children, respectively) [25, 26, 36]. In addition, our study also showed having children as a significant risk factor of HCMV infections during pregnancy (RR = 1.17). Considering the financial status, we noticed the lowest HCMV prevalence among women with the best status (53.9 vs. 58.3 % in women with very good vs. bad status, respectively). Fairly similar HCMV IgG seroprevalences were observed among pregnant women in Norway, where low family income was associated with higher CMV IgG seropositivity compared to high family incomes [62.9 vs. 53.8 %; odds ratio (OR) = 1.46, 95 % CI 1.00 to 2.12] [32]. However, in our study, the highest prevalence of HCMV infection was observed in pregnant women with good and average financial status (63.5 %), which may suggest the lack of influence of the economic status on HCMV infections in our cohort.

In the reported study, we also analyzed the occupational contact with children and blood transfusions as possible risk factors of HCMV infections. However, none of them was associated with HCMV prevalence rate. So far, the previously reported results related to the influence of occupational contact with children on HCMV prevalence rates were contrary to our observations [21, 37–40]. Based on those studies, we suggest that occupational contact with children is a population-dependent risk of HCMV infections. The observed discrepancies might originate from other factors, including hygiene behaviors and the age of children, cared for by the pregnant women included in the study [37, 41]. Accordingly to the role of transfusions in HCMV infections, we indicated a lack of any association between these factors investigated in our pregnancy cohort. This is similar to the relationship showed for multitransfused patients from Bangladesh [42]. No other papers have focused on transfusions as a risk factor of HCMV infections, although studies on HCMV prevalence among blood donors have been performed [43–45].

In this paper, we also analyzed the seroprevalence of anti-HCMV IgM antibodies. In the studied cohort, the IgM prevalence rate was 2.2 %. Similar prevalence rates were also reported for pregnant women in Finland (4.0 %), Australia (5.5 %), and France (5.7 %) [24, 27, 35]. In the case of the French cohort, the IgM prevalence rate of 5.7 % was characteristic for HCMV-IgG seropositive women, while for the whole study cohort, the IgM prevalence rate was 2.7 %, being similar to the prevalence observed among Polish pregnant women [27]. In addition, both our and French serological screenings were performed by means of the same laboratory tests [27]. In our study, we observed a significant association of the IgM prevalence with patient age, with changes in age-stratified prevalence rates similar to those observed for IgG antibodies. However, no current studies showed any association between age and the HCMV IgM prevalence rates [39, 40, 46]. We suggest that the relatively low IgM prevalence rates, reported in various populations, may have been responsible for the observed discrepancies. In the population of US pregnant women aged 12–49 years, the IgM prevalence rate was 3.0 %, but stayed relatively flat across the age groups [46]. Despite the fact that, in our cohort, age-associated differences in the IgM prevalence rates were observed, the lack of a significant age-related trend was fairly distinctive [46].

Similarly to other studies, no associations were found between the IgM prevalence rates and other risk factors, including education level, offspring, financial status, and the occupational risk of contact with children [24, 27, 47]. We suggest that the lack of relationships might be possibly caused by the relatively low seroprevalence of IgM in the study population (2.2 %; 28/1,250). It seems plausible that only a large cohort study might show the risk factors influencing the prevalence rate of the IgM antibodies. The IgM prevalence tended to be higher in pregnant women with transfusions in their history than in those without transfusion events (4.3 vs. 2.0 %). The lack of significance in the relationship observed in our study might have resulted from the small number of transfused women (3.9 %, 46/1,180). In Bangladesh, a significant increase was observed in the seroprevalence of IgM antibodies for multitransfused patients, showing transfusions as a possible risk factor of HCMV infections during pregnancy [42].

In comparison with other European pregnancy populations, the prevalence of IgG anti-HCMV in the Polish pregnancy cohort is high. Primary and vocational education, having children, and age above 36 years are most serious risk factors of HCMV infections during pregnancy. This indicates the primary prophylaxis to increase the awareness of the risks, related to HCMV infections, in pregnancy as an extremely important measure.
